# The Causes and Treatment of Baldness

**Published:** 1905-06

**Authors:** Henry Waldo

**Affiliations:** Senior Physician to the Bristol Royal Infirmary and in charge of the Department for Diseases of the Skin; President of the Dermatological Society of Great Britain and Ireland


### THE CAUSES AND TREATMENT OF BALDNESS.

BY

HENRY WALDO, M.D., M.R.C.P.,

*Senior Physician to the Bristol Royal Infirmary and in charge of the Department for Diseases of the Skin;*

*President of the Dermatological Society of Great Britain and Ireland.*

THERE is still some difference of opinion as to the cause of thinning of the hair. We all admit that early baldness may be the sequel of many fevers or any other general disease that interferes with nutrition. And there is also much more discrepancy of view regarding the etiology of coronal baldness, some maintaining that it is a natural infirmity of old age, in which the hair-bulbs decay prematurely, and corresponds to the loss of teeth or other degenerations of structure. In recent years our knowledge of this subject has been much increased by the researches of Unna, Sabouraud, and others. These investigators would lead us to look upon all forms of baldness as parasitic in origin. They say that thinning of the hair, whether general or beginning on the crown or at the temples and forehead (alopecia pityrodes), can be produced by a micro-organism. Seborrhœa oleosa is generally admitted to be at times physiological, but who is to say when the physiological limit ends. The only connection that seborrhœa oleosa has with disease of the skin is that it might form a culture-bed for the development of pathogenic organisms.

Sabouraud thinks the micro-bacillus of oily seborrhœa finds its way into the hair follicle and causes sebaceous hyper-secretion; then hypertrophy of the sebaceous glands; next, progressive papillary atrophy; finally, death of the hair. The late William Anderson admitted the fact of the coincidence of the hypertrophied sebaceous gland, the dilated hair follicle, and the bacillus-infested cocoon on the one hand, with the atrophic hair on the other as incontestable, but he thought it may still be doubtful whether the order of histological events is that assumed by the eminent observer. It was at least possible, he thought, that the atrophy of the hair-papilla in this form of alopecia is the primary condition, the hypertrophy of the sebaceous gland secondary—due, perhaps, to the diversion of blood supply from the dying structure to that which remains in full activity—and that the microbic invasion is explained by the lessened resistance implied by a lower vitality of tissue, the hair follicle, no longer filled by its normal contents, becoming a kind of empty purse for the dermal bacillus to play in. And he ventured to think that there are many reasons against the view that coronal baldness is either a seborrhœic or a parasitic affection in its origin. And in confirmation of this view he stated that coronal baldness starts at a definite spot, significantly coinciding with the site of the initial senile synostosis of the cranial bones (opposite the region of the parietal foramina), and usually sets in at a period contemporary with this first step in the sealing up of the brain casket, a spot that coincides, moreover, with the meeting-point of the most distal filaments of the three principal nerves supplying the scalp and with the anastomoses of the cranial blood capillaries most remote from the heart. In addition to these arguments, he said that it is a matter of common knowledge that this form of baldness is almost confined to men, while women, who are almost equally subject to the undoubtedly parasitic forms of alopecia, are spared. He also pointed out that it appears to be manifested earlier and more constantly in men of the educated classes than in those of the grades below, who wear less constricting, though more infective, headgear than their more favoured fellows. And he drew attention to its attacking persons of good physique, often the very pick of humanity, enjoying perfect health, rejoicing in good digestions, clear complexions, and highly-efficient nerve centres, and usually free from seborrhœa sicca, acne, and even the more visible manifestations of seborrhœa oleosa.

But, on the contrary, Sabouraud says that the essential element in seborrhœa oleosa is a specific micro-bacillus which produces, by virtue of the toxins which it generates, both the glandular hypertrophy and the papillary atrophy that constitute the anatomical lesions in coronal baldness as well as in alopecia areata. I think that there is no doubt that alopecia areata is sometimes contracted from domestic animals, especially the dog and cat. The current view is that alopecia areata is parasitic in origin in 95 per cent. of the cases; and if it is true (which Sabouraud affirms) that the same bacillus is the cause of alopecia areata and other forms of baldness, then it is fair to assume that coronal baldness may in some cases owe its origin to this cause. Now, when we come to discuss the origin of alopecia pityrodes, there is not much difference of opinion among dermatologists. In this form the thinning and baldness begins at the temples and forehead, and mostly takes the form of seborrhœa sicca. Crocker says that he does not consider there is any real etiological distinction between temporal and coronal baldness. He thinks fair people are more prone to seborrhœa sicca, and dark people to seborrhœa oleosa. He says it appears to run in families sometimes, and that it is a much more obstinate disease in the old than in the young; that there is often defect of health which is generally debilitating, and finally that in a large number of cases no cause whatever can be assigned for it, the patients being in robust health, and one can only assume a tissue proclivity which offers a favourable soil for the seborrhœic micro-organism. This reminds one of the early days following the discovery of the tubercle bacillus. We began to think that family history in phthisis pulmonalis was a bogey, but as years went on we very gradually found it necessary to reconsider our position, and who would now ignore the family history of tuberculosis?[1] Norman Walker says that those who suffer from seborrhœa (which, in his opinion, is the invariable cause of premature baldness), and yet preserve their hair, always have

[1] It had been laid down by the late Dr. Moxon that it was not so much the fact that the microbe and the man met, as the condition of the man when they did meet. And there might also be the anatomical condition of the skin as a factor, the peculiarities of soil.

an abundance of oily secretion on the scalp, and their hair early turns grey. He thinks the great predominance of baldness in the male sex is probably to be explained by their more frequent visits to the barber.

I have said nearly all I have to say regarding the causes of baldness, but there is a theory propounded by Parker in the *New York Medical Record*, 13th July, 1901, that I should like to mention. He says that alopecia is due to auto-intoxication with some substance derived from the lungs, owing to decomposition of organic material normally present in respired air when this air is retained in the air vesicles. The apices are the places of retention, owing to diaphragmatic breathing. This, he thinks, explains the comparative infrequency of baldness in women, in whom breathing is normally of the "costo-superior" type.

I have taken it for granted that seborrhœa in some form or other has a good deal to do with baldness, and I will add that Brooke's view of the pathology of this condition is "apparently a dermatitis caused by the presence of one or possibly several micro-organisms, and leading to a specific irritation of the fat-forming functions of the skin." I may say that Unna's view that the seat of the process is in the sudoriparous and not the sebaceous apparatus has not found, as Malcolm Morris says, general acceptance.

**Treatment.**—Of course, preventive measures are much the most satisfactory. It is sometimes possible to induce hair to grow when the disease has not been too long present, but it is even then more or less unsatisfactory. Assuming that bacilli are present they are then deep down in the hair follicle, and we are met with the same difficulty that occurs in tinea tonsurans, and accounts for the subjects of that condition, if they have not very great faith, going the round of the doctors, until the last one gets the credit for the cure, often after the fungus has expended itself. The scalp in the seborrhœic condition may look to an ordinary observer quite healthy, and yet there may be very marked funnel-shaped depressions round the hair follicles which are filled with fat. In some cases the seborrhœa, as Crocker says, may be limited to these depressions, the surface being clean, and although this cupping of the hair follicles occurs chiefly in severe cases, it may, he says, be partially or completely recovered from.

Now first, as regards washing and bathing. One hears ridiculous nonsense regarding washing the scalp, and especially from the female sex. There are ladies who are proud to boast that they have not washed their heads with soap and water for several months, perhaps years; they little know what an effective disinfectant is ordinary soap, and if it is superfatted it causes very little dryness of the hair. If there are well-marked signs of seborrhœa black soap is better, and if combined with spirits of wine and perhaps a little thymol, is a powerful remedy for good, and it should be repeated every day until all signs of the seborrhœa have vanished. If it were not for the concentration of hair-producing power in the scalp of women they would oftener be the victims of premature baldness. I think men and women should make a rule of washing their scalps with soap and water at least once a week, and especially after each visit to the barber, as it is here they are likely to become infected from the clipper or brushes or other materials. They should also occasionally soak their own brushes and combs in a 5 per cent. solution of carbolic acid.

An important preventive precaution is to induce an abundant blood supply to the scalp, which can be well carried out by simply moving the scalp upon the skull in different directions with the rough towel after the morning bath. It is well, too, at the same time to rub the ears, as it not only tends to prevent chilblains, but what is more important, it is said to ward off senile deafness, and it would be interesting to hear what aural surgeons think of this. Bathing, especially in the open sea, is thought to thin the hair, but I imagine that it never does any lasting harm. Much more important is it to warn people with perforated drum membranes that sea water is an irritant to the middle-ear, and that they run a great risk of speedy death from meningitis.

It is thought by some that hard hats are harmful, and especially if they fit tightly. I think, however, it is more important to see that the linings of the hats are clean, but at the same time a hat should not interfere with the circulation or innervation of the scalp, and it is better to have it perforated for ventilation. We were taught years ago that wounds of the scalp always healed easily as the parts were so vascular, but I suppose that surgeons of the present day have more fear of infection and greater difficulty in ensuring cleanliness in the scalp than in most other parts of the body, as it is such a nursery for germs. It is reported that a London surgeon on one occasion was able to trace a series of post-operative suppurations to a seborrhœic house-surgeon, the calamity coming to an end as soon as the seborrhœa was brought under control by appropriate treatment. It is thought, too, that seborrhœic patients are more prone than others to post-operative suppurations, probably through the agency of microbes retained within the dilated follicles of the skin in spite of the most sedulous cleansing.

When there is any tendency to seborrhœa it should be treated with a microbicide, but I am sorry to say not with much hope of curing it, for the tendency to recurrence dies only with the patient. Yet the more immediate complications of seborrhœa are readily checked by these remedies. As a palliative then—those who prefer to use some more or less greasy application, I would suggest half an ounce of precipitated sulphur made up with a pint of liquid paraffin, and applied to the scalp every morning: those who prefer a non-greasy microbicide, the same amount of sulphur to a pint of spirits of wine. I should not prescribe paraffin for females, as the danger of fire has to be thought of, and as a matter of experience ladies dislike putting anything greasy on their heads. People constantly ask for something that will make the hair grow. Well, the view we take is that the old stimulating remedies, even beef-marrow preparations, are unnecessary; for, as a rule, if the microbe and its immediate consequences are removed the hair is nearly always ready enough to grow. A daily lotion of acetic acid and resorcin, made up with eau de Cologne if you like, and rubbed well into the scalp with a piece of flannel, is as likely as anything else to soak down into the hair follicles without doing any harm. Nascent sulphur lotions are preferred by some, and then hyposulphite of soda and tartaric acid are used separately. Salicylic acid preparations often act well, and so does vasogen iodine. Where there is hyperæmia, other remedies of a more soothing character would be first used.

In conclusion, it appears to me that it may become fashionable to advise patients threatened with baldness to try the effect of X-ray treatment, for at the present time it is being used with good results in tinea tonsurans. A considerable discussion took place on this subject at the last meeting of the Dermatological Society of London, and the general opinion seemed to be that the method was now firmly established as the speediest cure for ringworm of the head. Many members had used it in numerous instances, and no member reported any untoward result, though all agreed that the greatest care must be used.

---

